# The outside control of subclavian-pulmonary anastomosis in postoperative period fallot's tetralogy patients

**DOI:** 10.1186/2197-425X-3-S1-A956

**Published:** 2015-10-01

**Authors:** RA Ibadov, HK Abrolov, NA Strijkov, DI Zhulamanova

**Affiliations:** Republican Specialized Center of Surgery Named after Acad. V. Vakhidov, Tashkent, Uzbekistan

## Objectives

Optimization of the tactics of intensive management of early postoperative period after regulated subclavian-pulmonary anastomosis in patients with Fallot's tetralogy.

## Materials and Methods

144 patients were treated after application of regulated subclavian-pulmonary anastomosis according to the technique devised in RSCS after V.Vakhidov within the period from 2005 to 2014, in the resuscitation and intensive care unit (RICU). Tourniquet regulator to proximal end of anastomosis was used in case of 78 patients and Fogarty catheter for volume dosing of shunt was used in case of 66 patients. By the time of surgery the age of patients ranged from 3 months to 22 years (mean 9,24 ± 3,21 years). 80 patients from total (57.69%) were male and 64 (42.31%) were female. All patients went through standard set of clinical examination: echocardiography (with detection of velocity of blood flowing through the subclavian-pulmonary regulated anastomosis), electrocardiography, chest X-rays (with special emphasis on assessing the degree of blood filling in the pulmonary circulation); cardiac monitoring: heart rate, blood pressure, central venous pressure, gas exchange parameters and deep oxygen status (pH, SpO_2_, pO_2_, pCO_2_, lactate); monitoring of hemoglobin, hematocrit levels; data of blood coagulation and protein fractions with optimized modes of ALV. The doses and amount of used cardiotonics was taken into consideration.

## Results

From overall 104 patients, 22 patients had hyperfunction of anastomosis: the picture of preedema was noted in 6 cases, and 10 patients had “managed” hypervolemia of the pulmonary circulation that manifested as: hemodynamic instability (MABP 75-80 mm Hg, HR -120-140 bpm, CVP - 100-140 millimeter of water), increase of SpO_2_ to 90,1 ± 1,2 together with poor values of deep oxygen status (A-a-205,1 ± 5,3 mm Hg, a/A-47,3% ± 1,4), rales revealed auscultatively, decreased transparency of lung fields in chest radiography. The all above mentioned values served as set of direct criteria for correction of hemodynamics by inflating the balloon of Fogarty catheter, and such achieving anastomotic constriction.

Restriction of anastomosis functioning allowed to achieve stabilization of hemodynamic indices (blood pressure 90 - 100 mm Hg), deep oxygen status (A-a and -230 mm Hg, a/A-30%, SpO_2_- 80% at FiO2- 40%), which were accompanied by disappearance of rales, improvement of radiological and echocardiographic data in dynamics with reduced duration of mechanical ventilation and time of staying in the ICU.

## Conclusions

The proposed tactics of intensive care in the early postoperative period after controlled subclavian-pulmonary anastomosis in patients with Fallot's tetralogy allows monitoring and active controlling of the volume of shunted through the anastomosis blood, thus helping to avoid development of hyperfunctioning of anastomosis and pulmonary edema.

